# Is Ambient Light during the High Arctic Polar Night Sufficient to Act as a Visual Cue for Zooplankton?

**DOI:** 10.1371/journal.pone.0126247

**Published:** 2015-06-03

**Authors:** Jonathan H. Cohen, Jørgen Berge, Mark A. Moline, Asgeir J. Sørensen, Kim Last, Stig Falk-Petersen, Paul E. Renaud, Eva S. Leu, Julie Grenvald, Finlo Cottier, Heather Cronin, Sebastian Menze, Petter Norgren, Øystein Varpe, Malin Daase, Gerald Darnis, Geir Johnsen

**Affiliations:** 1 The University Centre in Svalbard, 9171, Longyearbyen, Norway; 2 Faculty of Biosciences, Fisheries and Economics, UiT The Arctic University of Norway, 9037, Tromsø, Norway; 3 University of Delaware, School of Marine Science & Policy, 700 Pilottown Rd., Lewes, Delaware, United States of America; 4 Applied Underwater Robotics Lab, Depts of Biology and Marine Technology, Norwegian University of Science and Technology, N-7491, Trondheim, Norway; 5 Scottish Association for Marine Science, Scottish Marine Institute, Oban, Argyll PA37 1QA, Scotland, United Kingdom; 6 Akvaplan-niva A/S, Fram Centre, 9296, Tromsø, Norway; University of Western Australia, AUSTRALIA

## Abstract

The light regime is an ecologically important factor in pelagic habitats, influencing a range of biological processes. However, the availability and importance of light to these processes in high Arctic zooplankton communities during periods of 'complete' darkness (polar night) are poorly studied. Here we characterized the ambient light regime throughout the diel cycle during the high Arctic polar night, and ask whether visual systems of Arctic zooplankton can detect the low levels of irradiance available at this time. To this end, light measurements with a purpose-built irradiance sensor and coupled all-sky digital photographs were used to characterize diel skylight irradiance patterns over 24 hours at 79°N in January 2014 and 2015. Subsequent skylight spectral irradiance and in-water optical property measurements were used to model the underwater light field as a function of depth, which was then weighted by the electrophysiologically determined visual spectral sensitivity of a dominant high Arctic zooplankter, *Thysanoessa inermis*. Irradiance in air ranged between 1–1.5 x 10^-5^ *μ*mol photons m^-2^ s^-1^ (400–700 nm) in clear weather conditions at noon and with the moon below the horizon, hence values reflect only solar illumination. Radiative transfer modelling generated underwater light fields with peak transmission at blue-green wavelengths, with a 465 nm transmission maximum in shallow water shifting to 485 nm with depth. To the eye of a zooplankter, light from the surface to 75 m exhibits a maximum at 485 nm, with longer wavelengths (>600 nm) being of little visual significance. Our data are the first quantitative characterisation, including absolute intensities, spectral composition and photoperiod of biologically relevant solar ambient light in the high Arctic during the polar night, and indicate that some species of Arctic zooplankton are able to detect and utilize ambient light down to 20–30m depth during the Arctic polar night.

## Introduction

Light and the seasonality of light regime (also referred to as “light climate”) comprises irradiance and its spectral composition (i.e. spectral irradiance (E(λ), μmol photons m^-2^ s^-1^nm^-1^), polarization and photoperiod (hours of “illumination”) [1]. The light regime influences most biological processes, from primary production at the base of food chain through timing and phenology of reproduction, growth and foraging routines [2–5] to the distribution and numbers of organisms in space and time [6]. Especially at high latitudes, the seasonality of the light regime is at its most extreme, with extended periods of either midnight sun (summer) or polar night (winter). Accordingly, processes otherwise known to be regulated by available light might be assumed to be altered, paused or absent during periods of either continuous presence or absence of illumination. As an example, light is known to structure predator-prey interactions in aquatic environments through the prey-encounter of visually searching predators [7]. This is widely acknowledged in studies of lake ecosystems [8], but less studied and quantified in the marine environment (but examples include [9, 10]), despite its major effects when studied in models [11]. It generally remains unknown how important visual predation is at high latitudes during the polar night, but recent evidence indicates that the planktonic amphipod predator *Themisto libellula* is able to detect their calanoid prey even at 80°N during the darkest part of the polar night [12].

Above the polar circle, the period when the sun is below the horizon for a 24-hour period or more is called the polar night. The duration of the polar night and the corresponding irradiance in this period increase with latitude from south to north [13]. Due to the sun’s angle below the horizon, moonlight, and aurora, the polar night is not a homogenous dark period [14]. At 78°N 55’ in Kongsfjorden, Spitsbergen, the polar night lasts for 129 days each year, thus playing a significant role in the area’s light regime. Although this period was once thought to be void of biological activity, recent research (for a review, see [14]) challenges this assumption by presenting evidence of Diel Vertical Migration (DVM) throughout the polar night. However, despite evidence that DVM is usually considered to be tuned to an exogenous light cue [15, 16], there is no direct evidence that marine zooplankton would be sensitive enough to supposed extreme low light levels that characterize the high Arctic polar night. However, a behavioural study conducted on Calanoid copepods [16], a major component of the vertically migrating population during other times of the year [17], found that *Calanus* spp. were able to provide a phototactic response in the order of 10^–8^ μmol photons m^-2^ s^-1^ of blue light (peak emission 455nm with a band width of 23 nm) and 10^–6^ μmol photons m^-2^ s^-1^ of white light [16]. This finding is essentially the limit of our knowledge concerning visual systems in Arctic marine zooplankton, due in part to difficulties studying visual function in pelagic animals that commonly become disoriented when placed in small chambers [18].

Electrophysiological techniques allow animals’ visual capabilities to be assessed at the level of the photoreceptors in their eyes, as opposed to whole-animal behaviour that necessarily combines neural and motor responses and is dependent upon the behavioural assay used [19]. Electrophysiological recording is useful for measuring the spectral sensitivity of zooplankton vision, and has been particularly successful in studying comparative visual function in both shallow and deep-sea crustaceans [19, 20], including Antarctic euphausiid (krill) species (*Euphausia superba* and *Thysanoessa macrura*) [21] and the subarctic krill *Meganyctiphanes norvegica* [22]. Studies on the visual capabilities of krill with more northerly subarctic or Arctic distributions are lacking, despite evidence that these species perform visually-mediated behaviors, such as DVM, in the Arctic polar night [23]. The main objective of the present study, therefore, is to provide *in vivo* spectral sensitivity measurements for the eyes of a representative subarctic/Arctic zooplankter, the krill *Thysanoessa inermis*, and use them in conjunction with ambient light measurements (E_PAR_, 400–700 nm, Photosynthetic Active Radiation, μmol photons m^-2^s^-1^) and E(λ)) from high-sensitivity sensors and radiative transfer modelling to evaluate if zooplankton are able to utilize the available light at depth during the polar night.

## Materials and Methods

All sampling and field measurements were conducted between the 13^th^–27th of January 2014 and 2015 in Kongsfjorden, Spitsbergen at 78°55’N. The work was carried out according to the HMS guidelines of the local and national authorities for conducting fieldwork on Svalbard (see www.unis.no), and the project was entered into the Research in Svalbard (RiS) database with project number 6575. For projects registered in the RiS database and carried out in compliance with the Kings Bay AS, no specific permissions are required for marine work in Kongsfjorden. The work does not include protected or endangered species.

### Sky light field

Atmospheric light intensities were characterized by an irradiance sensor (IMO-PAR, In-situ Marine Optics, Perth, Western Australia) at sampling frequency of 5 Hz to measure E_PAR_ during 21–22^nd^ of January 2014. To enhance light sensitivity, the light sensor had been calibrated with its cosine diffuser removed, and was mounted on a tripod with its 20° aperture aligned to measure reflected light from a Spectralon plate that reflected 99% of the 400–700 nm PAR spectrum (SRT-99-050, Labsphere, NH, USA). Accordingly, the downward-facing sensor received 180° of diffuse reflected skylight [24]. Measurements were made from the Kings Bay Marine Laboratory (Ny-Ålesund, Spitsbergen) adjacent to Kongsfjorden. Artificial lights (buildings, lamp posts, etc.) near the light sensor were extinguished or screened, to minimize their impact on ambient light measurements. Irradiance (E_PAR_, μmol photons m^-2^s^-1^) was measured using a factory calibration coefficient after correcting for sensor noise measured in darkness (dark current) at environmental temperatures (averaging -3°C). No effect of changes in ambient temperature on dark noise was observed during the measurement period. Occasional artificial light sources (e.g. car lights, head lamps) were detected by the light sensor as high and distinct spikes, and were removed from the irradiance time series by applying a running median filter with a window size of 10 min and an overlap of 5 min. Adjacent to the E_PAR_ sensor was an all-sky camera [Canon EOS 5D Mark III with full size CMOS sensor (24x35 mm, giving a crop factor of 1)] equipped with a 8 mm fish eye lens (Canon EF zoom lens 8–15 mm, providing a 180° viewing angle at 8 mm) set to an constant ISO of 12800 (light sensitivity), aperture (f) of 4.5, white balance manually set to “daylight” and using the shutter speed as the only variable (ranging from 1.5–0.25 sec exposure time), in order to characterize relative irradiance and to detect different skylight scenarios occurring during corresponding E_PAR_ measurements. All-sky images were taken in RAW format every 30 minutes between 00:00 on the 21^st^ of January to 14:32 on the 23^rd^ of January 2014. Time series (21–22 Jan 2014) of E_PAR_ measurements and relative irradiance derived from all-sky camera shutter speed were in agreement and used to detect periods of maximum and minimum ambient light intensities.

### Underwater light field

The spectral irradiance from the atmosphere was used as an input into a radiative transfer model in order to characterize the underwater light field. Total incident spectral irradiance had 100% diffuse skylight (i.e. no direct solar/lunar light). Values were obtained with a QE Pro spectrometer (Ocean Optics, FL USA) calibrated for absolute irradiance measurement with a 200 μm entrance slit and 1000 μm optical fiber. Configuration of the spectrometer, fiber, and Spectralon reflectance plate was as described above for the E_PAR_ sensor. The light field throughout the water column was modelled using the HydroLight v. 5.2 RTE model. The model was set-up to provide a spectral output of 390–700 nm at 10 nm resolution (see S1 File for input and output data). Depth resolution was 1 m over the upper 75 m water column, assuming infinite bottom depth and no wind; mid-fjord depths are >300 m [23]. Index of refraction was constant for all wavelengths with a value of 1.340. IOPs required for the model included pure water absorption values [25], along with spectral absorption and scattering coefficients measured *in situ* at 6 Hz by an ac-9 absorption / scattering meter (Wet Labs, Oregon, USA) profiled through the water column at midday on the 23^rd^ of January 2015. Before use in the models, ac-9 data were processed for temperature and salinity effects [26], for scattering artefacts [27], and for instrument drifts since the last manufacturer’s calibration using pure water calibrations [28]. The model included inelastic radiative processes of Raman scattering and chlorophyll-*a* fluorescence [29, which we measured *in situ* in January 2014 to be low (~0.06 μg L^-1^ throughout the Kongsfjorden water column).

The purpose of this light model was to derive an underwater light field that could be related to zooplakton vision, particularly in the krill, *T*. *inermis*. However, the modelled full-spectrum underwater light field is not necessarily representative of light available to zooplankton visual systems due to the limited spectral sensitivity of their eyes [29]. To address this, modelled underwater light fields were transformed into "krill utilized photons" (E_krill_) by weighting the modelled scalar irradiance (E_o_; units of μmol photons m^-2^ s^-1^ nm^-1^) at each wavelength (λ) by the ability of *T*. *inermis* to detect light at that wavelength according to its normalized visual spectral sensitivity absorptance spectrum determined electrophysiologically (S(λ); see methods below):

$$E_{krill}=\int_{390}^{700}E_{o}(\lambda )S(\lambda )d\lambda$$

### Visual spectral sensitivity

Zooplankton were collected from Kongsfjorden with a 180 μm WP2 plankton net lowered vertically from a small boat down to 75 m, and subsequently retrieved. Once on the surface, the cod end was emptied into a black bucket under dim red light and organisms kept in darkness at 3–5°C for no longer than 3 days until used in electrophysiology experiments. Electrophysiology was done using electroretinogram (ERG) recording as described in detail elsewhere [28, 29]. Briefly, under dim red light (red LED head lamps) an individual *Thysanoessa inermis* (body length 12.3 ± 1.43 mm, standard error, n = 5 replicates) was isolated from plankton collections and prepared for electrophysiology by gluing its dorsal carapace and eye stalk to an acrylic support with cyanoacrylate adhesive. The specimen was submerged in a temperature-controlled water bath within a light-tight Faraday cage, and then an epoxy-insulated tungsten microelectrode (127 μm diameter, A-M Systems, WA USA) was positioned subcorneally by micromanipulator under dim far-red light (Schott RG630 longpass filter, NY USA). Temperature in the water bath at the position of the animal's eye was 4.5°C (± 0.6 SD, n = 5 replicate krill preparations) throughout the duration of the experiments, and all individuals survived the experimental protocols lasting 6–22 h. Spectral sensitivity of the *T*. *inermis* eye was measured in 5 different individuals using the criterion response method (for detailed methods see [30–32]). Spectral sensitivity data were modelled [33] to predict the best-fit rhodopsin visual pigment and its specific absorbance given a photoreceptor length of 56 μm determined from semi-thin sections of resin embedded *T*. *inermis* sampled from these same collections. Spectral sensitivity data determined by ERG recording from *Thysanoessa inermis* is presented in S1 File.

## Results

### Sky light field

**Fig 1:**
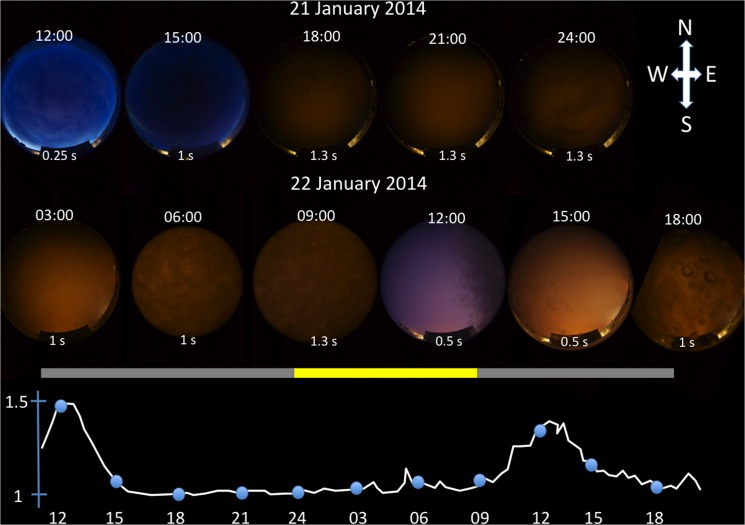
All-sky pictures from Ny-Ålesund 21st and 22nd of January 2014. Time of day is indicated on each picture and corresponds to a blue circle on the graph (bottom; E_PAR_, 400–700 nm, Photosynthetic Active Radiation) showing the absolute value of ambient light at that point in time in units of x10^-5^ μmol photons m^-2^s^-1^. The rectangular bar indicates the time of day when the moon is below (grey) and above (yellow) the horizon, and aligned with the time-scale on the irradiance graph below. On each picture the exposure time is given—all pictures were taken with the same ISO setting. The time-lapse camera and the light sensor were located next to each other

During the time of the investigations in Kongsfjorden the sun was between 9.4° and 8.3° degrees below the horizon during midday (solar noon), hence characterized as *nautical polar night* [14]. The moon was only above the horizon between 23:51 the 21^st^ of January and 08:34 the 22^nd^ of January, during which weather conditions were cloudy (Fig 1). Any change in E_PAR_ due to moonlight was below the detection limit for the instrument; all cyclic changes in E_PAR_ were therefore due only to sunlight. At noon on both days the weather conditions were clear. Using an all-sky camera and the E_PAR_ sensor in concert, the ambient irradiance ranged between 1–1.5 x 10^–5^ *μ*mol photons m^-2^ s^-1^ at different time points characterizing day and night situations under different weather conditions (Fig 1).

### Visual spectral sensitivity of krill

**Fig 2:**
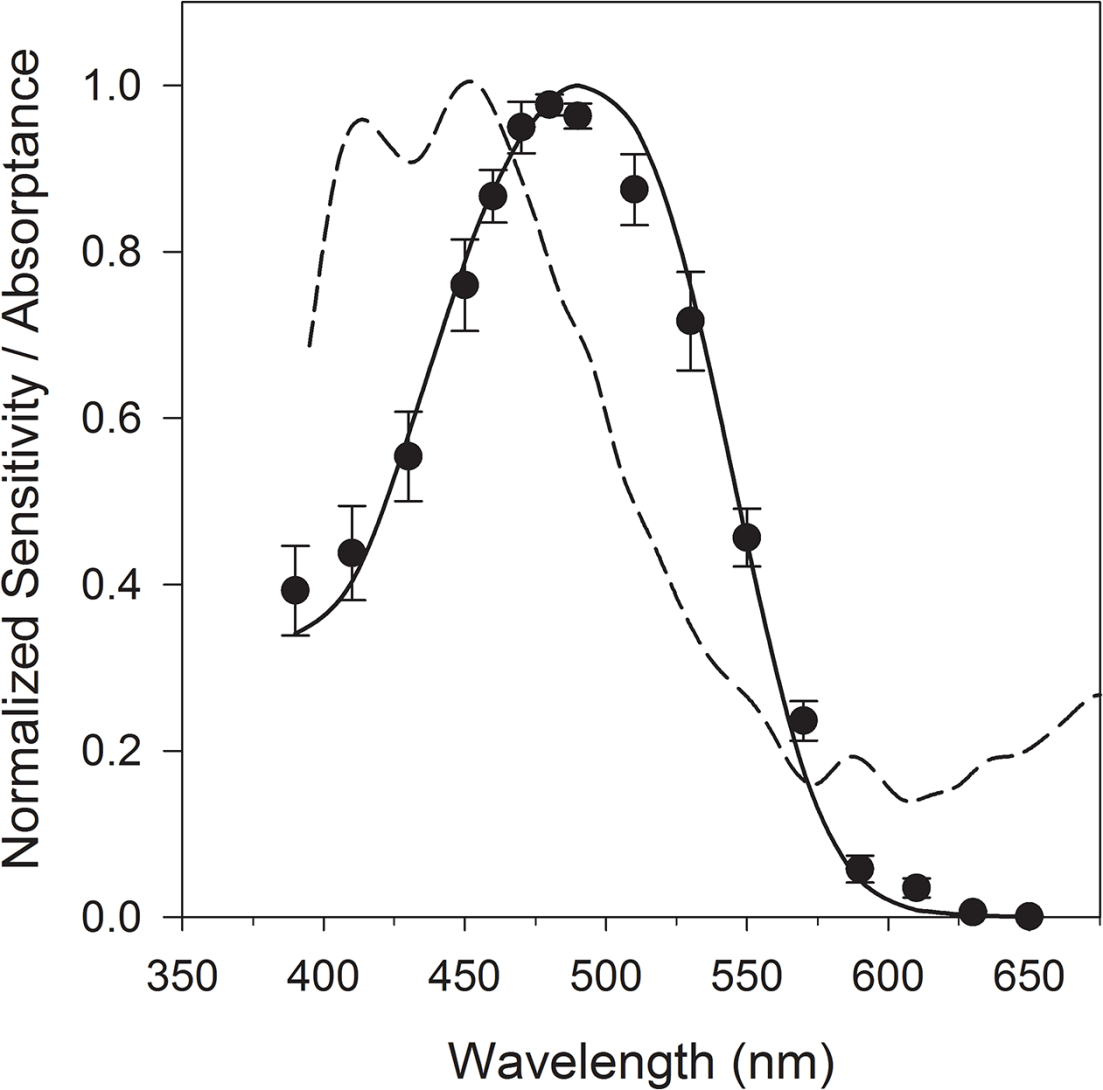
*Thysanoessa inermis* visual spectral sensitivity and spectral composition of skylight in the polar night (dashed line). Spectral sensitivity data are means (± standard error, n = 5) with the best-fit rhodopsin absorptance (solid line, λ_max_ = 492 nm). Spectral irradiance measured in air at noon on the 21^st^ of January 2015 has been normalized to its peak; integrated absolute irradiance as PAR for this measurement was 1.3x10^-5^ μmol photons m^-2^ s^-1^.

Electroretinogram recording from *T*. *inermis* eyes yielded a visual spectral sensitivity curve that peaked in the 470–490 nm region (Fig 2). These data were best-fit by a rhodopsin visual pigment with maximum absorbance (λ_max_) of 492 nm, and a specific absorbance of 0.010 μm^-1^ (residual sum of squares = 0.0267).

### Underwater light field

**Fig 3:**
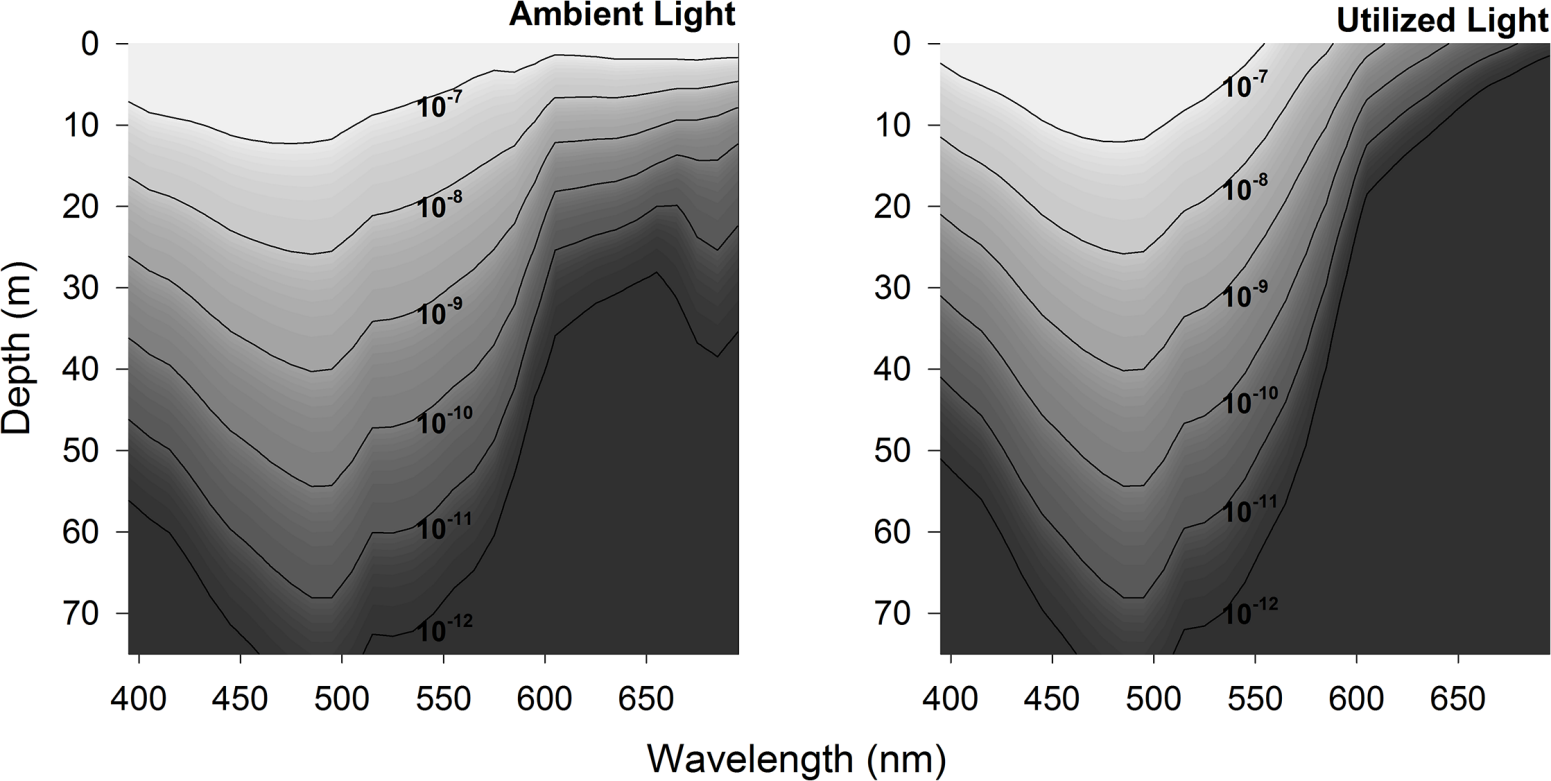
Modelled underwater spectral light field in Kongsfjorden at midday under clear sky conditions. Contours show the ambient underwater light as scalar irradiance (Ambient Light, left panel) and krill-utilized photons (Utilized Light, right panel). For both panels, light is expressed in units of μmol photons m^-2^ s^-1^ nm^-1^, derived from a radiative transfer model as described in the Materials and Methods.

Skylight spectral irradiance at solar noon on the 21^st^ of January 2015 give an E_PAR_ of 1.3x10^-5^ μmol photons m^-2^ s^-1^ was similar to that measured by the E_PAR_ sensor at the same time in the previous year (i.e. 1.5x10^-5^ μmol photons m^-2^ s^-1^). When these spectral irradiance data were propagated through the water column by radiative transfer modelling, maximum transmittance was in the blue-green region, with a 465 nm peak at 10 m depth shifting to 485 nm by 30 m (Fig 3, left panel). Expressing modelled light levels in terms of krill-utilized photons resulted in a 485 nm peak by 10 m; this maximum transmittance continued with depth due to the krill spectral sensitivity maximum in this wavelength region (Fig 3, right panel). Also notable is that wavelengths >600nm, while present in ambient underwater light field, are poorly detected by the krill eye.

## Discussion

### The light regime

The light regime is a major ecological factor. In the Arctic, with a highly variable annual light:dark cycle, light is responsible for controlling the timing of numerous ecological processes and behaviors, and therefore a vulnerable point for the ecology of many Arctic pelagic organisms in a changing climate [1, 34–38]. Recent work in Kongsfjorden (Ny-Ålesund, Spitsbergen) has shown that ecologically relevant light levels must be present during the winter "polar night" period, when the sun is below the horizon over the diel cycle [2]. Our light measurements in air indicate a dynamic change in light regime with respect to intensity and spectral composition. There is a clear photoperiod with a short (~5 hour photophase) and highest irradiances observed at solar noon when the sun is at its highest elevation below the horizon. Variation in spectral irradiance can be visualized by the all-sky camera images with differences in colour (clear sky versus cloud cover). Periods of clear sky show the blue part of the visible spectrum dominating, which is also evident in our spectral irradiance measurements. This is of high significance for blue to blue-green sensitive marine organisms, using this light for a range of biological processes [5, 15, 16].

Underwater light sources include both atmospheric light (moon, aurora, starlight and diffuse sun light) as well as underwater bioluminescence [2, 12 23, 39–41]. As the Arctic warms, current levels of atmospheric light will increase with reduced winter ice cover [38, 42], while the resulting underwater light field will likely be complex resulting from the pattern and process of ice melt [43]. More information is needed on the photosensitivity of Arctic marine organisms in order to understand how this changing light regime will impact pelagic ecology. The modelled underwater light field in Kongsfjorden during winter has a major spectral peak in ambient light transmission at blue-green wavelengths (peak transmission at 465–485 nm). This modelled underwater light field is spectrally similar to previous reports [44] for this location in May, a time of year before fresh water run-off from glaciers starts (often by the first week of June) due to elevated temperatures and midnight sun (April-September at this latitude) with corresponding variation of sub-surface light climate through IOPs by phytoplankton blooms (Chl a), coloured Dissolved Organic Matter (cDOM) and Total Suspended Matter (TSM) [1, 3, 4, 45]. Further work is needed to determine how variable the underwater light field in Kongsfjorden may be during winter with intensity and spectral changes, for example with moonlight or cloud cover apparent in our all-sky imagery, as well as other times of the year, but our model results appear to characterize typical winter conditions.

### The ability of zooplankton to detect light

This study determined visual spectral sensitivity of an abundant zooplankter in Kongsfjorden throughout the year, the Arctic krill *T*. *inermis* [46]. This species performs DVM, with daytime residence at depth and ascent to the surface at night [47]. Our electrophysiological experiments with *T*. *inermis* suggest its visual spectral sensitivity in blue (492 nm maximum) is similar to other polar (Antarctic) and boreal vertically migrating krill species studied with similar techniques (487–492 nm maxima; [21]. This spectral sensitivity is well-aligned with the major spectral peak in deeper Kongsfjorden water at 485 nm, and could facilitate photon capture of atmospherically-derived light at those wavelengths.

**Fig 4:**
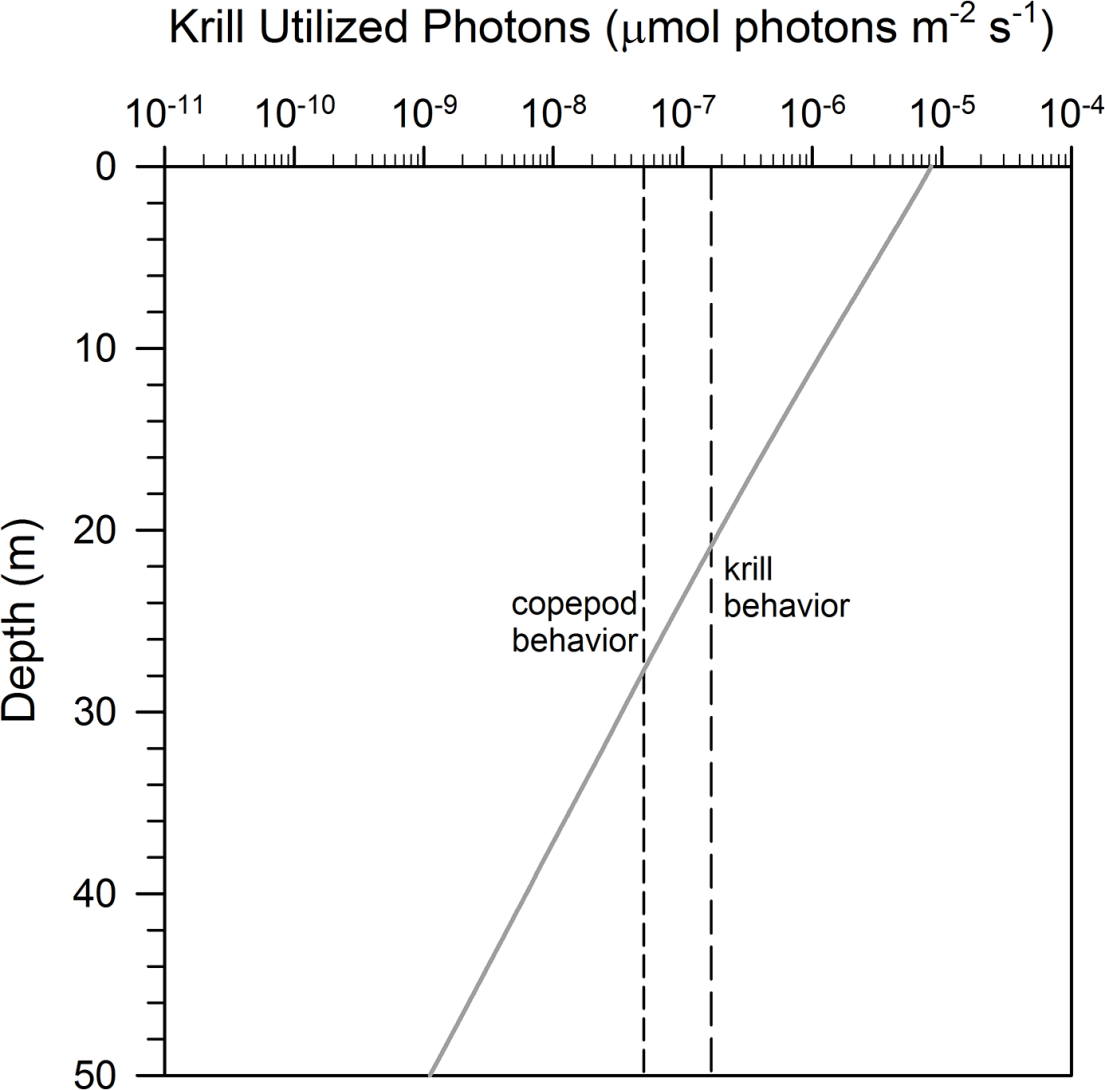
Spectrally-integrated midday light in Kongsfjorden as related to zooplankton thresholds for light-mediated behavior. Spectrally-integrated irradiance as krill-utilized photons is plotted as a function of depth (grey line). Lower visual thresholds determined behaviorally in previous studies with blue broadband light are plotted as vertical lines for krill *Meganyctiphanes norvegica* (krill behavior, medium dash, Myslinksi et al. 2005) and copepods *Calanus* spp. (copepod behavior, short dash, Båtnes et al. 2013).

It is informative to examine the underwater light field in the polar night as it appears to zooplankton more generally, and this can be done by using the spectral sensitivity of the *T*. *inermis* eye to correct the modelled scalar irradiance into utilized photons. When this is done, and compared to thresholds for light-mediated swimming behaviour in the krill *Meganyctiphanes norvegica* [22], it is clear that light perception by krill extends to over 20 m depth (Fig 4). Similarly, the threshold for light-mediated swimming in another abundant zooplankter in Kongsfjorden, the copepod *Calanus* spp. [16], is sufficient to enable light detection to near 30 m depth. Cyclic atmospheric light at these depths may serve as a diel cue for DVM during winter [2]. However, it may be that endogenous rhythms are the proximate cue driving DVM in deeper water during the polar night [2, 23, 39]; such rhythms are involved in DVM of other zooplankters from lower latitudes [29] and our study has shown evidence of a photoperiod from this location during the polar night that could entrain such rhythms.

Beyond 30 m depth in Kongsfjorden, where light is nearing the lower limit for zooplankton vision, their eyes may still be useful for light-mediated trophic interactions, specifically for detection of bioluminescence. In apparent darkness, bioluminescence will appear as a bright flash against a dark background. Bioluminescence has been characterized in Arctic waters [48, 49] and is abundant in Kongsfjorden during winter [23]. In this way, zooplankton vision during the polar night can serve both to maximize photon capture at shallow depths and aid in bioluminescence detection deeper in the water column.

### Outlook and conclusions

Here we present the first quantitative characterisation, including absolute intensities and spectral composition, of biologically relevant ambient light in the high Arctic during the polar night. Further, we have documented a biologically relevant photoperiod at a time of year when a photoperiod has generally been assumed to be absent or non-detectable. In the sky, the ambient sunlight varied during the study period between 1–1.5 x 10^–5^ μmol photons m^-2^ s^-1^, with peak values at noon in clear weather and with the moon below the horizon. Regarding zooplankton and their ability to detect and respond to these light levels, our results—by combining the *in situ* spectral measurement of ambient skylight, radiative transfer modeling, and *in vivo* spectral measurement of the ability of *Thysanoessa inermis* to detect light—we conclude that zooplankton are likely to detect light from the upper 20–30m of the water column during the time of exploration. This is the first study that unequivocally suggest that zooplankton are able to cue upon ambient light during the dark polar night, and is as such important for studies on zooplankton behaviour, including both patterns of diel vertical migration and trophic interaction.

## Supporting Information

S1 File Spectral sensitivity data determined by ERG recording from *Thysanoessa inermis* (datashown in Fig 2, and used to weight modeled light in Figs 3 and 4) and input / output data forthe light field model (Hydrolight). (XLSX)
